# From lipid detection to biological meaning: raising standards for oxylipin signaling

**DOI:** 10.1016/j.jlr.2026.101145

**Published:** 2026-09-11

**Authors:** Lingshuang Wu, John T. Shuster, Judith A. Simcox

**Affiliations:** 1 Department of Biochemistry, University of Wisconsin-Madison, Madison, WI, USA; 2 Howard Hughes Medical Institute, University of Wisconsin-Madison, Madison, WI, USA; 3 Cell Molecular Biology Graduate Program, University of Wisconsin-Madison, Madison, WI, USA; 4 Medical Scientist Training Program, University of Wisconsin-Madison, Madison, WI, USA

## Abstract

The field of oxylipin research has outpaced the development of standards and guidelines. As these signaling lipids gain attention in inflammation, metabolism, and tissue repair, the field must confront a central challenge: many proposed pro-resolving mediators are difficult to measure and interpret. A recent publication by Schebb and colleagues calls for stronger evidence, from analytical detection to endogenous function.

Metabolic and inflammatory diseases are rising globally, with rates of each doubling from 1990 to 2020. The rapid rise in disease prevalence has galvanized the search for environmental drivers, with lipids gaining traction as major regulators of both disease development and progression (1, 2). Specifically, seed oils and dietary polyunsaturated fatty acids (PUFAs) are of emerging interest because they are substrates for oxylipins. Oxylipins are a class of signaling lipids that regulate broad biological functions, including insulin resistance, immune cell recruitment and activation, and vascular tone. Beyond the availability of PUFA substrates, oxylipin levels are controlled by enzymatic activity, cell state, tissue context, pharmacology, and oxidation. The complexity of oxylipin regulation makes the connection between dietary PUFA intake and inflammation difficult to establish (3, 4, 5).

Specific oxylipins, including lipoxins, resolvins, protectins, and maresins, have been proposed to function as specialized pro-resolving mediators (SPMs) that help guide the transition from inflammation toward tissue repair and homeostasis (Fig. 1). SPMs have been proposed as therapeutic targets for metabolic and inflammatory diseases, but before their roles can be defined, there is a critical need to enhance the analytical quantification and data analysis pipelines for these oxylipins. Pro-resolving oxylipins remain one of the more contentious areas of lipid-mediator biology (6, 7). The controversy around the SPM is not only biological; it is also driven by the technical challenge of identifying and measuring low-abundance oxylipins (8). ELISAs often lack sufficient specificity, while liquid chromatography-mass spectrometry (LC-MS/MS) methods may struggle to resolve isomers. Furthermore, sample oxidation during analysis can de novo generate lipid species that may appear biologically meaningful. Strengthening the analytical and biological evidence underpinning SPM interpretation is especially important in the context of assigning biological and clinical meaning.

**Fig. 1:**
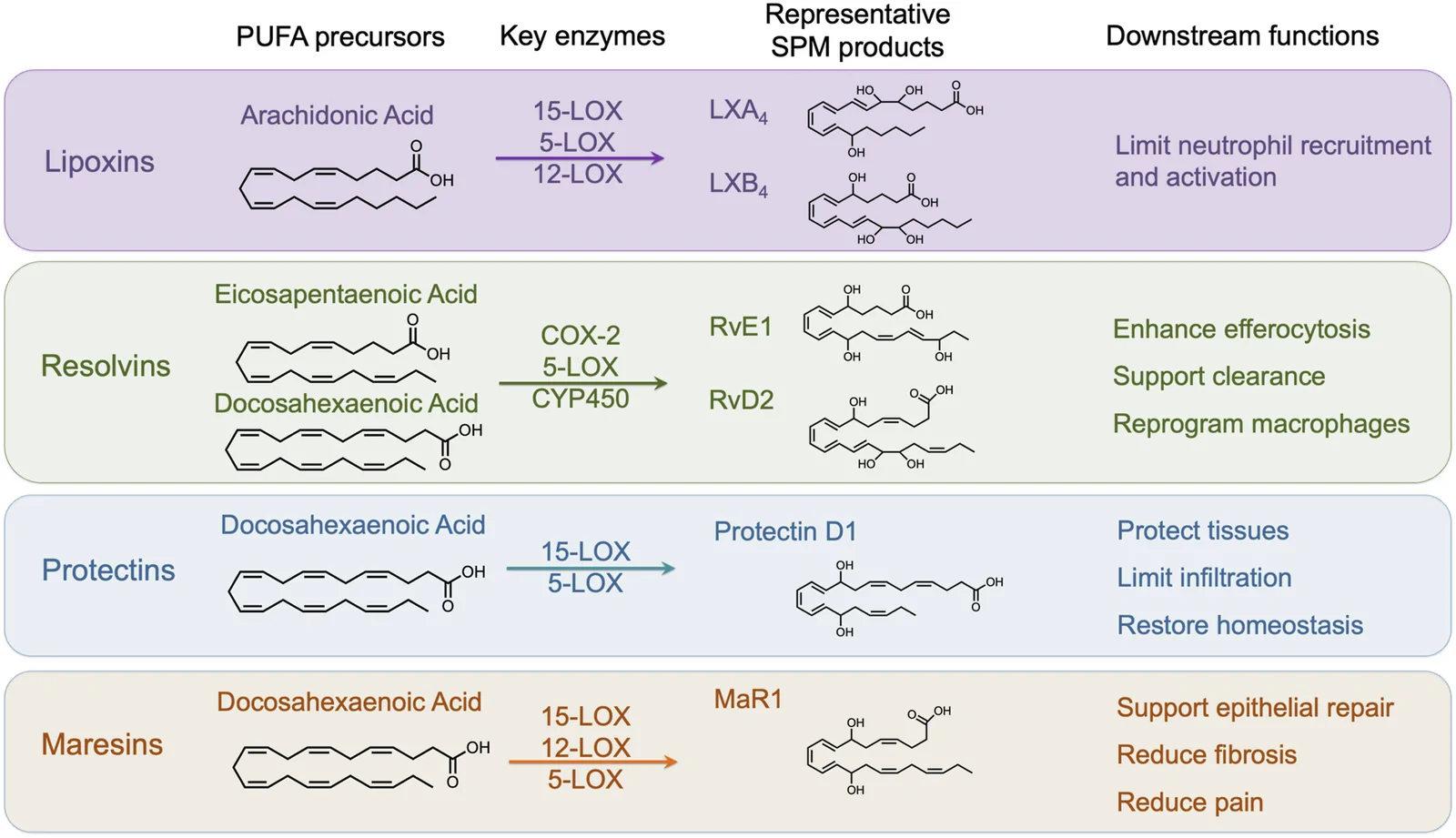
Major families of specialized pro-resolving mediators (SPMs): From precursor to functional outcomes. Specialized pro-resolving mediators are proposed to be generated from polyunsaturated fatty acid precursors through enzymatic pathways involving lipoxygenase, cyclooxygenase, and cytochrome P450 activities. Lipoxins are derived from arachidonic acid, E-series resolvins from eicosapentaenoic acid, and D-series resolvins, protectins, and maresins from docosahexaenoic acid. Representative downstream functions include reduced neutrophil recruitment, enhanced efferocytosis, macrophage reprogramming, tissue protection, epithelial repair, and resolution-associated signaling.

The review by Schebb and colleagues, focusing on validation of SPM analytical quantification (9), is particularly timely given that oxylipin biology is expanding faster than the standards and guidelines used to interpret analytical measurements. The authors center the discussion around three questions that are conceptually simple but often difficult to answer: (1) did we really detect the molecule, (2) can cells produce it endogenously, and (3) is the endogenous molecule present at levels sufficient to drive biological function? These questions sound basic, but for low-abundance lipid mediators, they are precisely where major issues of reproducibility between groups emerge.

The first question of detection is particularly important. When Schebb and colleagues reanalyzed raw data from published studies, the authors demonstrated that these prior observations were reporting noise rather than verifiable signal. A strong assignment requires more than a reported transition; it requires a visible ten-point chromatographic peak, established signal-to-noise criteria, and chiral separation when stereochemistry is claimed. The same level of standardization should apply to biosynthesis of SPMs -- utilizing stable isotope tracing to track precursor incorporation into downstream products directly. Prior studies focus on SPM synthesis from purified enzymes or precursor supplementation, and while this work demonstrates that these reactions are chemically possible, it does not mean cells make the SPM species endogenously. Finally, exploring the endogenous function of SPMs will require mechanistic studies including perturbations of the pathway, rescue, and assessment of phenotypic outcomes. The review emphasizes that partial evidence is insufficient to define a molecule as an endogenous SPM. To call a molecule an SPM, the field needs the full set of evidence: identity, biosynthesis, abundance, target engagement, and function (Fig. 2).

**Fig. 2:**
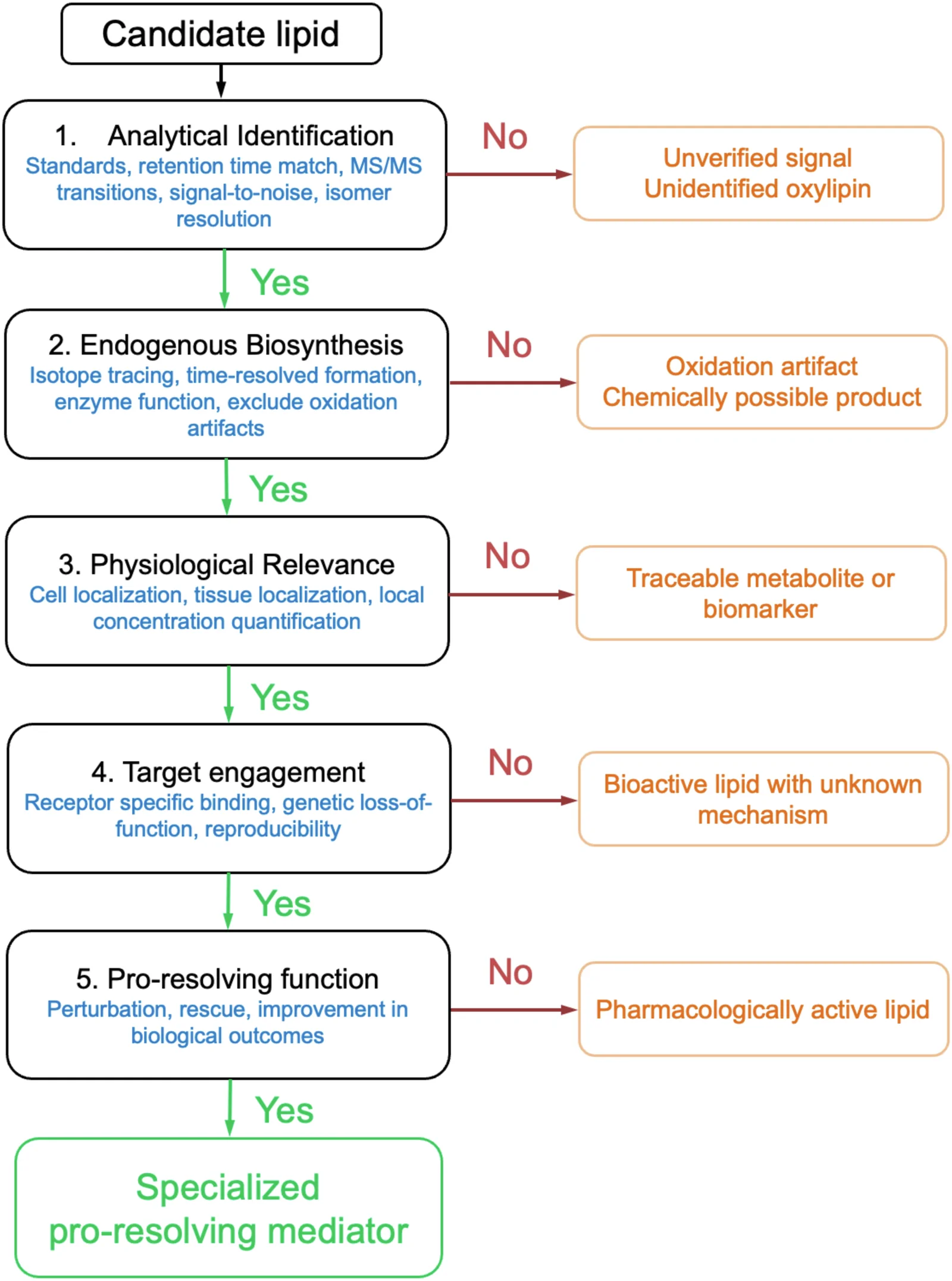
Decision map for defining an endogenous specialized pro-resolving mediator. A candidate lipid must be rigorously identified, shown to arise from unperturbed endogenous biosynthesis, detected at relevant abundance, linked to target engagement, and demonstrated to contribute to resolving inflammation.

## A call to action for the oxylipin field

The broader contribution of this review moves the SPM debate from a question of individual molecules to a question of field standards. Schebb and colleagues provide a practical roadmap for future work to match claims to evidence. The review encourages the field to prioritize fewer molecules, validate them more deeply, and match the strength of biological language to the strength of the evidence.

The field will only move forward with collective work from three sources: journal assessment, data repositories, and strong leadership from laboratory heads. For studies that claim detection of low-abundance oxylipins, journals should require raw data during peer review, not only after publication. This will allow the editors and reviewers to access the chromatographic evidence behind the central claims. Public data repositories are equally important. Availability alone is not enough; repositories need to preserve true raw files when possible, maintain data integrity, and store enough metadata for meaningful reanalysis. At the same time, they must be simple enough for laboratories to use consistently. Laboratory leaders have a responsibility to ensure raw data availability, report results transparently and inspect chromatograms carefully as routine practice. In this sense, the review highlights a broader principle for lipidomics: methods are not static. The culture of the field needs to shift toward continual reassessment and open data as new standards, instruments, software, and biological questions emerge.

Several existing resources can help the field move in this direction. The International Lipidomics Society Oxylipin Analysis Interest Group has already shown what community leadership can accomplish by coordinating technical recommendations for LC-MS/MS oxylipin analysis (10). At the same time, method development studies continue to show that these recommendations will need to evolve with instrument design, chromatographic materials, and data-processing choices (11). The next step is to transition these recommendations into living documents that are updated regularly, linked to repository requirements, and translated into practical checklists for authors, reviewers, and editors. The Lipidomics Standards Initiative (LSI) provides a foundation for harmonizing reporting and data quality expectations. LIPID MAPS remains essential for lipid nomenclature, structure annotation, and classification in a field where naming conventions can be confusing. Skyline and related data-analysis platforms can support transparent processing, peak inspection, and reproducible quantification when used with appropriate standards and manual review. Looking forward, what is needed is not only more resources, but stronger expectations that these resources become part of routine oxylipin reporting.

By establishing a clear oxylipin framework, the review changes what should count as convincing evidence in oxylipin biology. A disease-associated lipid feature may be a useful biomarker. A confidently detected oxylipin may be an endogenous metabolite. A lipid whose production depends on defined enzymes may be a regulated pathway product. However, establishing that a molecule is an endogenous pro-resolving mediator carries a substantial burden of proof: rigorous identification, endogenous production, relevant concentrations, and a direct link to a targeted pathway. This distinction is constructive rather than restrictive. By raising the evidentiary standard, the field can move from assigning names to lipid signals toward exploration of biological function.

## Conflict of interest

The authors declare the following financial interests/personal relationships which may be considered as potential competing interests.

J. A. S. is an HHMI Freeman Hrabowski Scholar. William H. Peterson Fellowship in Biochemistry (to J. T. S).
